# 3D atlas of the human fetal chondrocranium in the middle trimester

**DOI:** 10.1038/s41597-024-03455-1

**Published:** 2024-06-13

**Authors:** Markéta Kaiser, Tomáš Zikmund, Siddharth Vora, Brian Metscher, Igor Adameyko, Joy M. Richman, Jozef Kaiser

**Affiliations:** 1 Central European Institute of Technology, Brno University of Technology, Brno, Czech Republic; 2 The Life Sciences Institute, The University of British Columbia, Vancouver, Canada; 3 Department of Evolutionary Biology, University of Vienna, Vienna, Austria; 4 Department of Neuroimmunology, Center for Brain Research, Medical University of Vienna, Vienna, Austria; 5 Department of Physiology and Pharmacology, Karolinska Institutet, Stockholm, Sweden

**Keywords:** Cartilage development, Bone development, X-ray tomography

## Abstract

The chondrocranium provides the key initial support for the fetal brain, jaws and cranial sensory organs in all vertebrates. The patterns of shaping and growth of the chondrocranium set up species-specific development of the entire craniofacial complex. The 3D development of chondrocranium have been studied primarily in animal model organisms, such as mice or zebrafish. In comparison, very little is known about the full 3D human chondrocranium, except from drawings made by anatomists many decades ago. The knowledge of human-specific aspects of chondrocranial development are essential for understanding congenital craniofacial defects and human evolution. Here advanced microCT scanning was used that includes contrast enhancement to generate the first 3D atlas of the human fetal chondrocranium during the middle trimester (13 to 19 weeks). In addition, since cartilage and bone are both visible with the techniques used, the  endochondral ossification of cranial base was mapped since this region is so critical for brain and jaw growth. The human 3D models are published as a scientific resource for human development.

## Background & Summary

The knowledge of fetal anatomy is crucial for understanding developmental processes and their potential abnormalities in the human body. Genetic or environmental exposures during prenatal development can lead to anomalies in the skull and face that can cause serious malformations. However, studying human embryos is a challenge as access to human conceptuses is limited in many parts of the world. The experimental work is usually done on small mammals such as rodents or zebrafish as the model organisms^1–3^. The main previous work that looked at 3D development of the human craniofacial complex consisted of intricate 3D reconstructions of serial sections. However, these studies were limited to just one part of the head, typically the mandible. When specimens were older than 12 weeks, only one side of the mandible could be studied^4–7^. However, this histological method is destructive and larger specimens cannot be studied whole. Most human collections focus on the embryonic period up to 56 days gestation^8^. Optical Projection Tomography has also shown potential in human embryo imaging and creating a digital atlas^9^. However, the method is limited by penetration depth and only Carnegie stages 12–23 (i.e. 30–56 days post conception) could be imaged.

Other methods are needed to image the whole head. X-ray computed microtomography (microCT) is a promising method. The advantages of microCT are that a variety of samples sizes can be studied (from metres down to micrometres length scales), that there is no destruction of tissue and that the accurate 3D relationship of complex structures like the head can be preserved without distortion. MicroCT technique was used for the imaging of human embryos from the Kyoto Collection of Human Embryos (Kyoto, Japan)^10^. However, in this study only mineralized bones could be seen. More scans of the Kyoto collection were carried out with Magnetic Resonance Imaging (MRI)^11–17^. These MRI scans offered valuable insights into 3D morphology. MicroCT with staining has shown a great progress in last decade regarding the soft tissue examination in humans^18,19^. In our previous work, we showed the potential of microCT combined with staining for studying human foetuses^20^. Now, we exploit this potential for creating a full 3D atlas of human embryos’ chondrocranium in the middle trimester.

Unlike facial and calvarial bones, the cranial base is initially a cartilaginous structure, gradually being converted to bone via endochondral ossification. As described for the facial bones, the cranial base bones also develop from multiple ossification centers. In our work, we focus also on the ossification of the chondrocranium and we map the ossification on 3D model during the growth. The sphenoid, in particular, is formed by the eventual merging of multiple small bilateral bones. Consequently, the cranial base undergoes dynamic changes during the fetal period. Disturbances in synchondrosal growth and closure timing can affect overall craniofacial growth. For example, arrested growth or premature closure of the spheno-occipital synchondrosis correlates with severe midface hypoplasia in patients with craniosynostosis syndromes (Apert, Crouzon, Pfeiffer)^21^. Premature closure of the basi-occipital synchondroses can lead to foramen magnum stenosis in patients with Achondroplasia, resulting in severe neurological morbidity^22,23^. Despite the significance of the cranial base synchondroses, prenatal growth and development of this region remains poorly documented. The microCT scans in our collection encompass the ossification of chondrocranium, providing a great resource for studying this region. This has not been mapped using modern methods in which the earliest signs of skeletal tissue can be detected with more sensitive methods. 3D models are an important addition to the standards used during ultrasound assessment of fetal growth^24^ and could permit a full developmental study to be carried out for bones, cartilages and soft tissues of the skull during the middle trimester.

## Methods

**Fig. 1:**
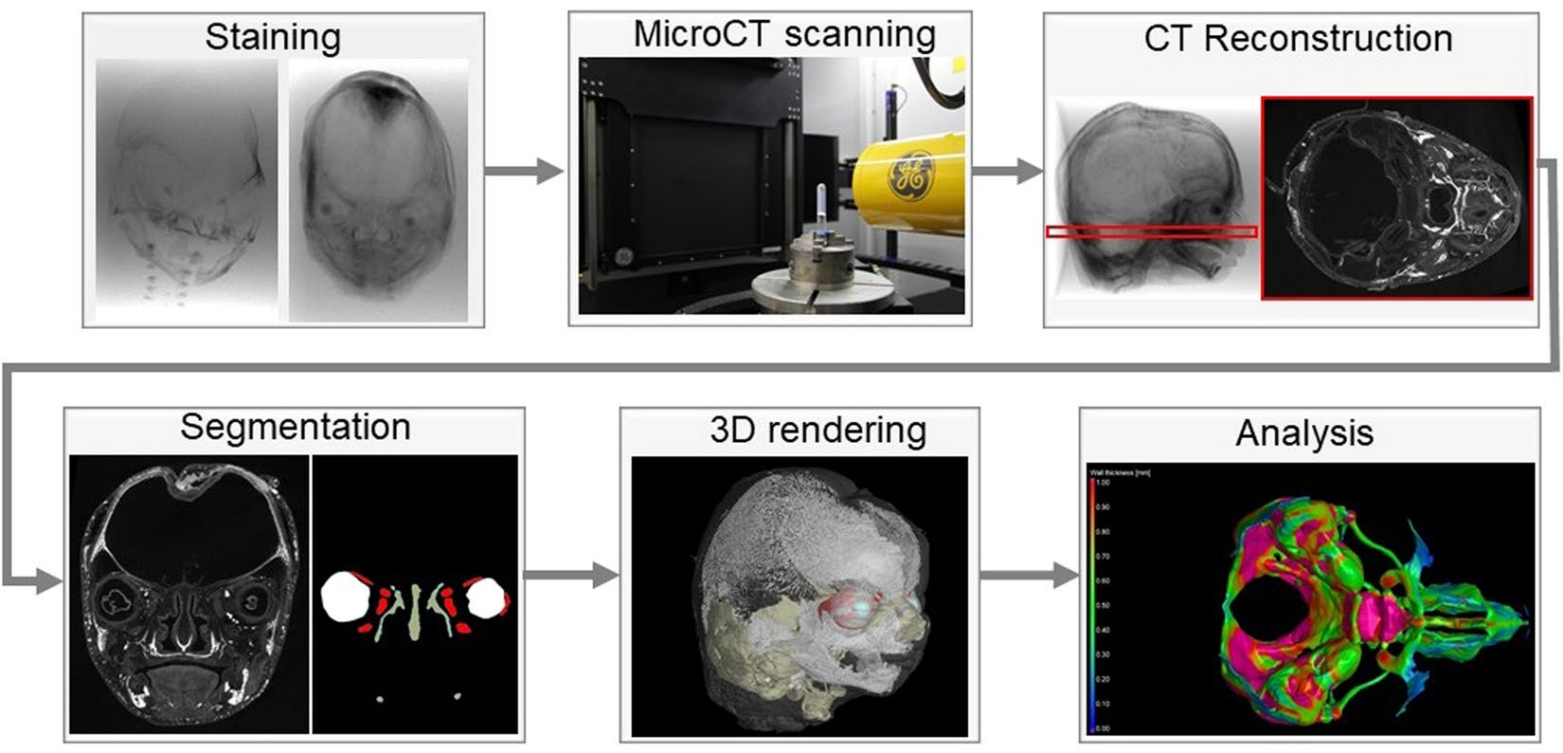
Experimental workflow. The samples were stained, scanned by microCT and after CT reconstruction, tomographic data were segmented. Segmented 3D models can serve for visualisation as well for further analysis.

In this work, 3D imaging of human embryos from 13 to 19 weeks of gestation is presented by contrast-enhanced X-ray computed microtomography (microCT). The existing collection at the University of British Columbia, Canada, was accessed. The samples were stained by phosphotungstic acid (PTA) and the segmentation focused on cartilaginous structures in the head. Such 3D models can be further analysed by volumetric 3D analyses or mathematical modelling. A schematic overview of the experiment is shown in Fig. 1.

### Sample collection

The existing collection at The University of British Columbia was accessed (UBC Ethics approval #H22-02933). The collection was obtained as part of a research project that was carried out by Dr. Virginia Diewert, professor Emerita of UBC. Dr. Diewert had funding from the provincial grant agency, BC Health Research Foundation in the mid 1980’s and in that grant, she had arranged to obtain pathological specimens of elective or spontaneous terminations carried out in the local Women’s Hospital (BC Women’s Hospital). In the original UBC ethics protocol written by Dr. Diewert, the fetal specimens were described as been collected in a non-invasive manner or consisting of materials that would normally be discarded. This was acceptable to the clinical research ethics board at UBC at that time. These terminations were legal and continue to be legal under British Columbia law. In addition, no personal identifiers were provided at that time. No post hoc identification is possible since the DNA is degraded. The waiver of informed consent for the publication of the data was requested for the new protocol. This manuscript will be the first publication since the new ethics protocol was approved at UBC by the clinical research ethics board at UBC (first approval was in 2008, new approval in 2022).

### Staining

For staining, PBS was changed to 30% methanol in water for 2 days. The samples were dehydrated in methanol series (50%, 70% and 90%), every concentration for 2–3 days. The staining was done by 1% PTA (Sigma-Aldrich P4006-100G) in 90% methanol for 3–4 weeks, dependent on the size of the head. Subsequently, the samples were rehydrated in methanol series (90%, 70%, 50% and 30%), again 2–3 days for each concentration. After that, the samples were mounted in 1% (aqueous) low-melting agarose gel for microCT scanning.

### X-ray computed microtomography measurement

The tomographic measurement was realized using X-ray microCT system GE Phoenix v|tome|x L 240 (Waygate Technologies / Baker Hughes Digital Solutions GmbH, Wunstorf) equipped with a 180 kV/15 W maximum power nanofocus X-ray tube and a high-contrast flat panel dynamic detector 41|100 with 4000 × 4000 pixels and a pixel size of 100 × 100 μm. The exposure time was 600 ms in 2500 positions over 360°. Three projections were captured in each position and an average of the signal was used to improve the signal-to-noise ratio. The microCT scan was carried out at 90 kV acceleration voltage and with 220 μA X-ray tube current. The beam was filtered by a 0.5 mm-thick aluminum filter to avoid beam hardening artifacts. The isotropic voxel size of obtained volumes depended on the size of the head and is stated in Table 1.

**Table 1:** List of the scanned samples including resolution and volume of cartilage and bone in the chondrocranium.

Developmental stage	Resolution (µm)	Volume (µm^3^)	
		Cartilage	Ossification
13 weeks	25	798	34
14 weeks	25	1020	84
15 weeks	29	1449	200
16 weeks	32	1916	461
17 weeks	36	2194	294
18 weeks	36	2068	1108
19 weeks	35	1553	1052

### Data processing

The tomographic reconstruction was performed using GE phoenix datos|x 2.0 software (Waygate Technologies / Baker Hughes Digital Solutions GmbH, Wunstorf, Germany). Reconstructed slices were imported to software Avizo 7.1 (Thermo Fisher Scientific, Waltham, MA, USA) for semi-automatic segmentation as described in our previous work^25^: Chondrocranium was outlined by the operator in every 3^rd^ to 5^th^ slice depending on the complexity of the structure and the rest was calculated by linear interpolation between manually outlined slices. The segmented chondrocranium was transferred to polygonal mesh (STL format) and transferred to VG Studio MAX 3.5 (Volume Graphics GmbH, Heidelberg, Germany) for further visualisation and analysis. The chondrocranium was split into two regions of interest: cartilage and ossification based on the greyscale value of tomographic data.

## Data Records

The generated data are available in the BioStudies with accession number S-BSST1227 (https://www.ebi.ac.uk/biostudies/studies/S-BSST1227)^26^. Seven samples (13, 14, 15, 16, 17, 18 and 19 weeks) were scanned and each sample has its own folder containing original tomographic data and segmented 3D models. Reconstructed tomographic slices are in the tiff format for all samples. The folder with the 3D model contains an STL file of chondrocranium and bone, separately. The segmented masks in the tiff format can be found in the separate folder containing binarized masks of all samples. Apart from the folder corresponding to each developmental stage, the output of this work containing all 3D models is provided in small data format including interactive 3D PDF^27^.

## Technical Validation

**Fig. 2:**
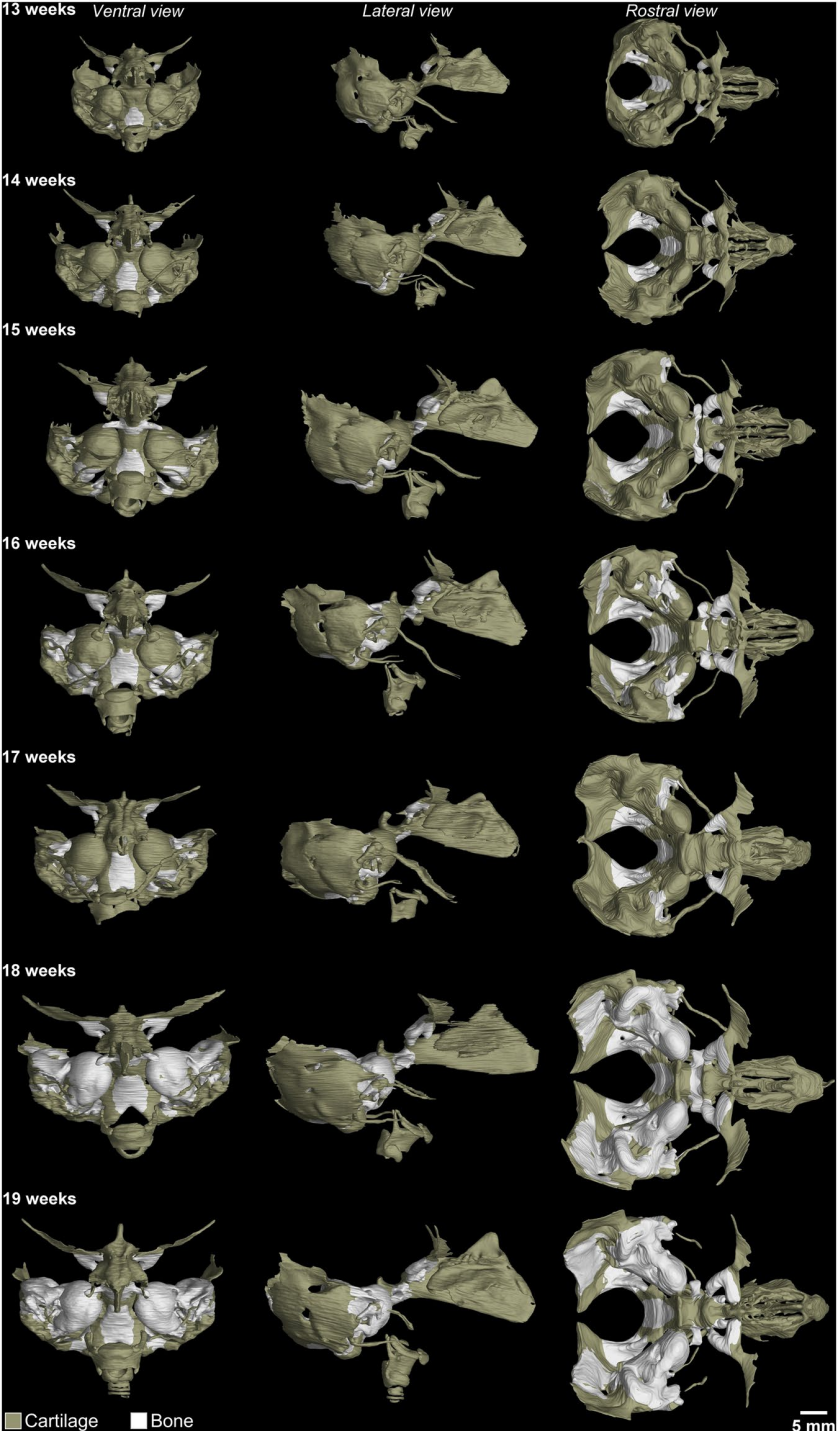
3D reconstruction of human embryos’ chondrocranium 13–19 weeks. Ventral, lateral and rostral view on the 3D models.

**Fig. 3:**
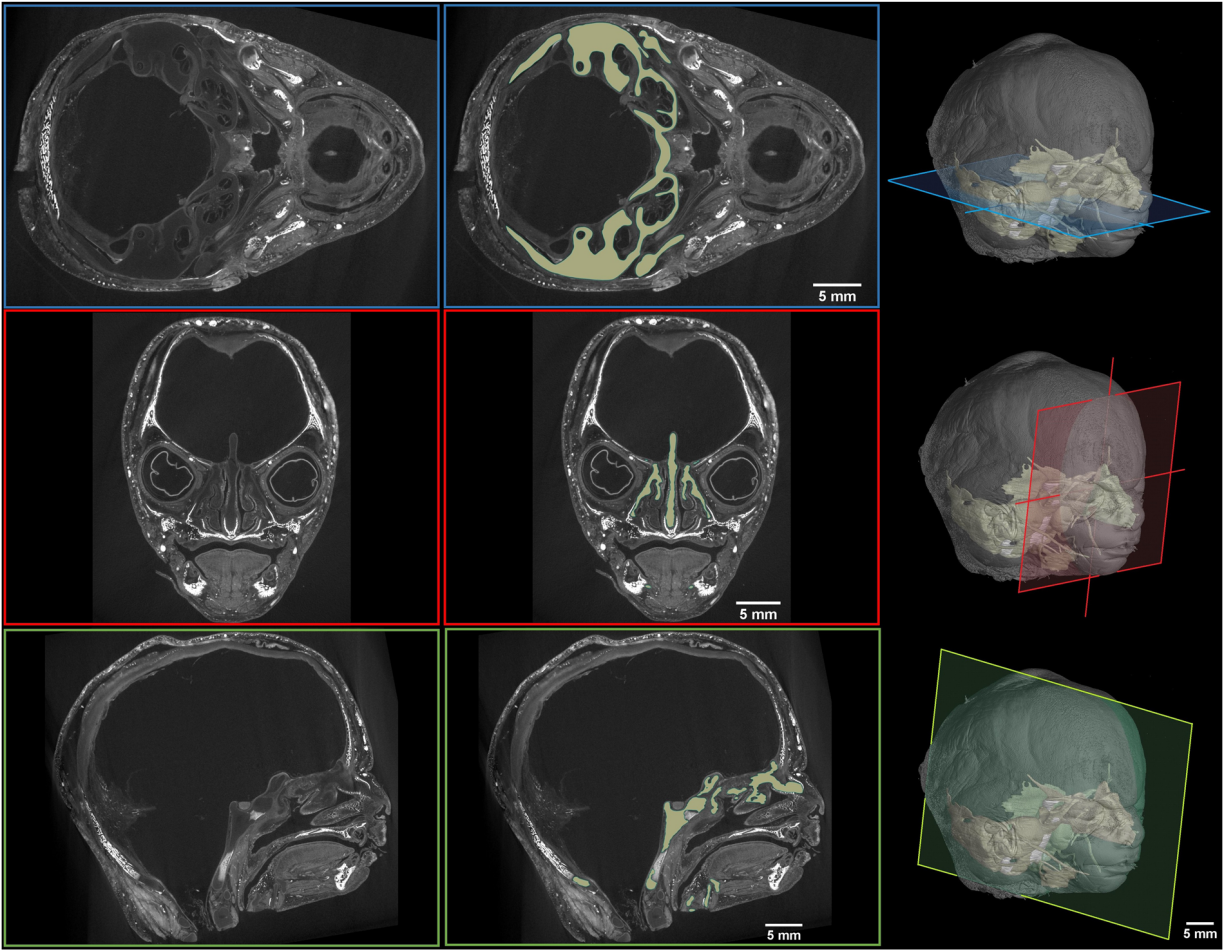
Validation of the segmentation on 14 weeks old sample. Color planes on the 3D models indicate the position of the CT slices: Transversal (blue), frontal (red) and sagittal (green) cross-section. Each cross-section show original raw CT slices (left) and the segmented chondrocranium (marked by brownish color in the right CT slice).

The knowledge on 3D anatomy of chondrocranium was gained by extensive studies on mouse models^28–31^. The anatomy of chondrocranium has never been seen before in 3D. The presented 3D models show the larynx, Meckel’s cartilage with ear ossicles, inner ear, cranial base or nasal capsule (Fig. 2)^26^. To be sure about appearance of cartilage/bone in microCT data stained by PTA, microCT slices were compared with stained histological sections by Alcian blue (for cartilage) and Alizarin red (for bone) on mouse embryos^28^. The segmentation was done manually in transverse view, to validate the accuracy of outlined structures, the segmented parts were checked also in frontal and sagittal section as shown in Fig. 3. The embryos heads used for scanning were cut in the neck area: For this reason, the segmentation of larynx differs among the samples. However, we tried to get maximum from the available samples and thus, the larynx was segmented despite the fact, it is not complete in all samples.

## Usage Notes

The reconstructed tomographic slices can be viewed in any imaging software supporting tiff data format. We recommend use of free ImageJ^32^ for viewing raw data. The 3D models can be opened directly in Microsoft Paint 3D available in Windows or free software as Meshlab^33^ or Blender^34^. For more detailed exploring and further analysis, we recommend specialized tomographic software; During our work, we used VG Studio MAX (Volume Graphics GmbH, Heidelberg, Germany) and Avizo (Thermo Fisher Scientific, Waltham, MA, USA).Other free software is available, including Drishti^35^ and Dragonfly (Object Research Systems, Inc., Montreal, QC, Canada).

### Supplementary information

Interactive 3D PDF

## Data Availability

No custom code has been used. All relevant information are described in the Methods section and Usage Notes.
