# Supplementary material for: 3D atlas of the human fetal chondrocranium in the middle trimester
Source: Sci Data. 2024 Jun 13;11:626. doi: 10.1038/s41597-024-03455-1 (PMC11176318; doi:10.1038/s41597-024-03455-1)
Supplement: Supplementary file 1 — Interactive 3D PDF [file 41597_2024_3455_MOESM1_ESM.pdf]

Interactive 3D reconstruction of human embryos’ chondrocranium

Developmental stages

13 weeks | 14 weeks | 15 weeks | 16 weeks | 17 weeks | 18 weeks | 19 weeks

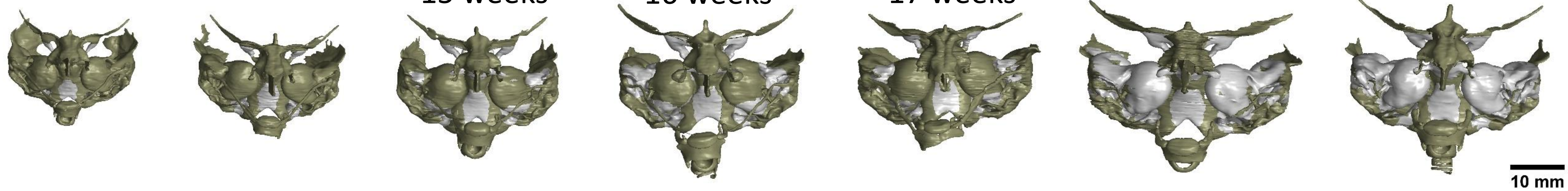

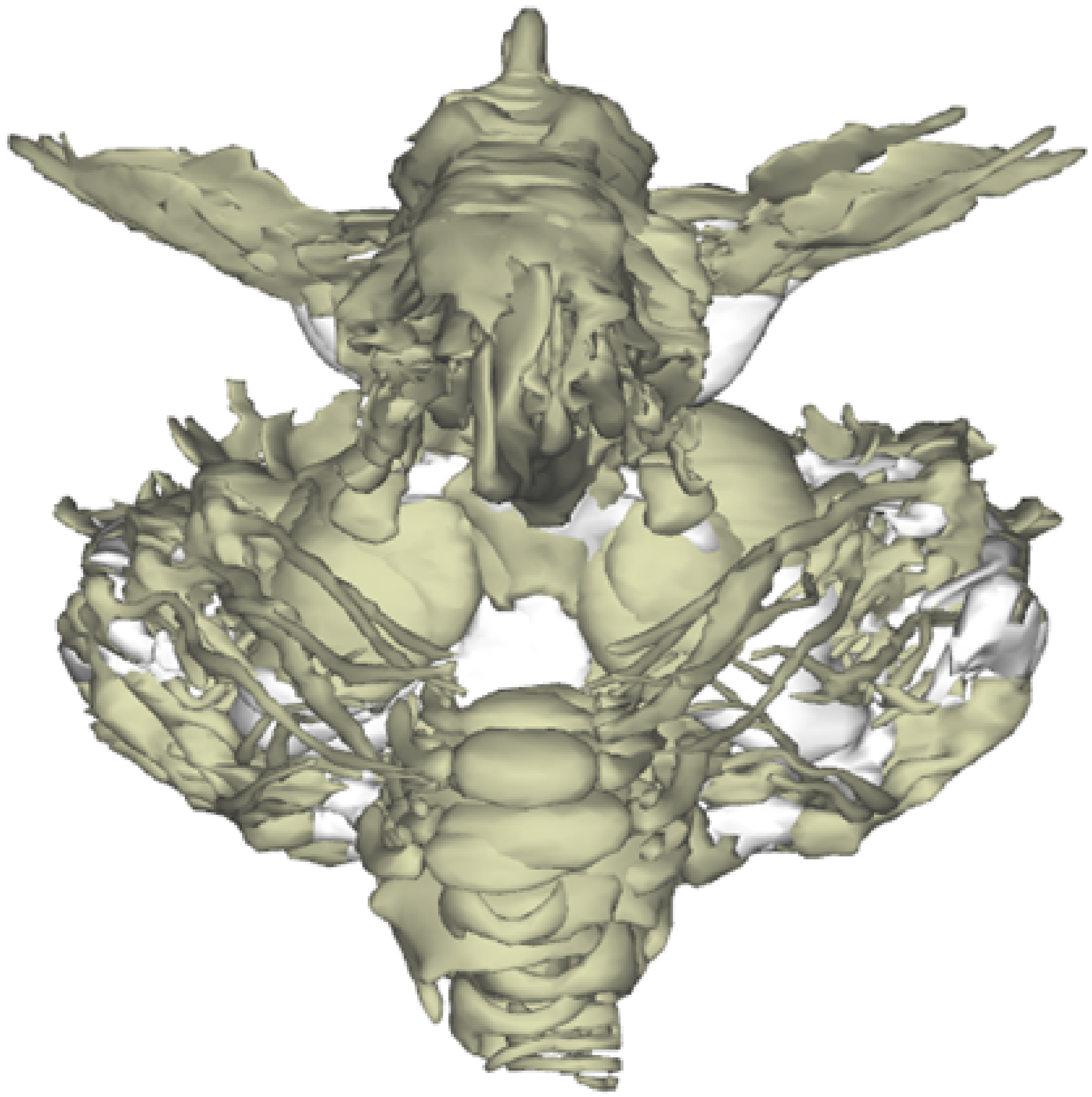

Technical note

To view interactive PDF files, you need to use the free standard Adobe Reader®/Acrobat Reader DC (www.adobe.com/downloads/). If you see a warning message in the yellow dialog box on the file opening, you can activate the content by pressing the button “Options”, otherwise you can enable 3D data for all documents in the preferences dialog box (Edit / Preferences / 3D & Multimedia / check the box “Enable playing of 3D content”).
